# Digital personas of AI use in nursing education: a latent class analysis of learning behaviors and academic engagement

**DOI:** 10.3389/fmed.2026.1859093

**Published:** 2026-08-14

**Authors:** Majed M. Aljabri, Bandar S. Alharbi, Endale Alemayehu Ali

**Affiliations:** 1 Community and Psychiatric Mental Health Department, College of Nursing, King Saud University, Riyadh, Saudi Arabia; 2 Department of Public Health and Primary Care, KU Leuven, Kapucijnenvoer, Leuven, Belgium

**Keywords:** academic performance, AI use in education, artificial intelligence, latent class analysis, nursing education, student engagement

## Abstract

**Background:**

Artificial intelligence (AI) is quickly revolutionizing higher education; however, there is high heterogeneity regarding the adoption of AI amongst the students. In the context of nursing education, where successful learning, critical thinking skills, and professional accountability are crucial, it becomes essential to understand how the students interact with the AI applications. The current study was conducted to determine the AI-use profiles among nursing students and how they relate to academic outcomes and demographics.

**Methods:**

We used a cross-sectional survey among nursing students. AI use for learning purposes was assessed using four binary indicators capturing use for understanding concepts, summarizing content, drafting assignments, and language support. Study engagement was measured using selected items from the Online Student Engagement framework and collapsed into three ordinal categories. We assessed academic performance using three self-reported items. We performed a latent class analysis to identify distinct student profiles based on AI use and engagement indicators. Multinomial logistic regression examined associations between demographic factors and class membership. Multiple linear regression assessed differences in academic performance across classes.

**Results:**

The best model was a three-profile solution with a good classification accuracy. The profiles included strategic engagers, moderate users, and passive or low engagers. As expected, strategic engagers were characterized by high levels of engagement with all kinds of behaviors studied, whereas Passive used consistently reported low levels of engagement. Usage indicators of AI revealed little variation among classes. The performance scores of strategic engagers were significantly better compared with those of passive users (β = 2.39, 95% CI = 2.08–2.69). No significant association was found for demographic characteristics.

**Conclusions:**

Nursing students can be categorized into distinct profiles based on their patterns of AI use and study engagement. Our findings showed that study engagement, but not AI use alone, was the key factor associated with academic performance. These findings highlight the importance of promoting effective learning strategies alongside AI integration. Educational interventions should focus on guiding students toward strategic and responsible use of AI to enhance learning outcomes.

## 1 Introduction

Artificial Intelligence (AI) technology has fast evolved from being an exclusive technology area into one of the key elements in modern-day universities (1). The introduction of AI-based tools in all the major subjects has significantly changed the approach to gathering data, completing homework, and learning in general (2). According to a recent study, students use AI tools by both emotional factors like perceived enjoyment and cognitive evaluations, including ease of use and perceived usefulness (3). This phenomenon represents the general digitization process that takes place in the field of education. As AI tools become increasingly integrated into educational settings, researchers have begun examining their potential benefits and challenges for teaching and learning outcomes.

In this developing environment, the topic of integrating AI into higher education became popular among educational scholars. According to meta-analysis, the adoption of AI technology has the potential to provide numerous benefits in education, specifically for nursing program, by providing efficient and high quality nursing service (4). In contrast, other scholars have raised concerns that excessive reliance on AI may undermine independent thinking, academic integrity, and critical reasoning skills (5). These contrasting findings suggest that the educational value of AI may depend less on the technology itself and more on how students incorporate AI into their learning processes. Therefore, understanding patterns of AI use among students is essential for determining when AI functions as a learning support tool vs. when it may hinder deeper learning. The integration of AI in nursing education have particularly important and need special attention. Nursing training requires not only theoretical knowledge but also clinical reasoning, decision-making, and ethical judgment. Evidence specific to nursing education remains inconsistent. While a recent systematic review reported improvements in learning attitudes, educational effectiveness, and clinical competencies associated with AI-supported learning (6), other studies have reported more nuanced findings. For example, Khlaif et al. (7) found that trust in AI was positively associated with academic performance, whereas greater AI competency was unexpectedly associated with lower performance. Such findings indicate that the relationship between AI and educational outcomes is complex and may be influenced by differences in students' learning behaviors, levels of engagement, and purposes for using AI.

From a theoretical perspective, the educational impact of AI can be understood through student engagement frameworks, which posit that learning outcomes are influenced not only by the availability of educational resources but also by the extent to which students actively participate in cognitive, behavioral, and emotional learning activities (8). AI tools may support learning by facilitating access to information, providing personalized explanations, and assisting with academic tasks. However, the benefits of these tools might not be the same across students. This is because, learners differ in how they incorporate AI into their study routines, the purposes for which they use AI, and the degree to which AI complements rather than replaces active learning processes. Consequently, educational outcomes may depend on distinct combinations of AI use behaviors and study engagement patterns. This perspective is important to identify subgroups of students with similar behavioral profiles, which may provide a more comprehensive understanding of AI's role in learning than examining individual factors in isolation.

Understanding AI use separately may provide an incomplete picture of its educational impact. Academic engagement is widely recognized as a main determinant of learning success. Because, it reflects the degree to which students actively participate in educational activities, invest effort in their studies, and remain cognitively involved in learning tasks. Although AI tools can support learning by providing rapid access to information, explanations, and academic assistance, their effectiveness is likely influenced by the extent to which students remain actively engaged in the learning process. Students who use AI strategically to complement their learning may achieve different outcomes from those who rely on AI passively (9). Consequently, examining AI use, academic engagement, and academic performance together offers a more comprehensive framework for understanding how AI contributes to educational outcomes. Beyond concerns related to academic integrity, algorithmic bias, and overreliance on AI, literature has also highlighted broader risks associated with excessive digital engagement, including concepts such as digital obesity and “brain rot,” which describe potentially adverse cognitive and behavioral consequences of prolonged digital technology use (10, 11).

Despite an increasing interest worldwide, there are still a lot of inconclusive evidence. Most studies have centered on a specific component of AI use, such as perception or attitude, or its impact in isolation rather than on capturing the whole picture of how AI technology is incorporated into students' learning behaviors (12–14). Regionally, research on AI in education has expanded in recent years, particularly in Asia and emerging digital economies, where rapid technological adoption has accelerated integration into university systems. However, empirical study conducted in the Middle Eastern region is relatively scarce and primarily centered around general attitudes and institutional preparedness rather than actual behavioral practices among students. The current literature indicates that culture, institutional policies, and familiarity with AI technology play a role in influencing adoption and utilization trends (15). Within the Middle Eastern region, Saudi Arabia provides a particularly important setting for investigation because of its ongoing digital transformation and increasing investment in AI-enabled educational technologies. In Saudi Arabia, where there is an increasing trend toward embracing digital transformation and the Vision 2030 plan (16), efforts to incorporate new technologies, such as AI, have become more pronounced in universities (17). Although some empirical studies that used Saudi respondents have emerged recently to examine perceptions about AI among educators and students, only limited studies conducted. Notably, researchers in this area stress the importance of context-specific studies to examine the actual use of AI by students in learning, rather than perceptions about it. Studies concentrating on Saudi nursing students and their use of AI in relation to studying are limited in number. Importantly, these studies emphasize the need for context-specific research that captures how students actually use AI in their academic practices, rather than relying solely on self-reported attitudes or intentions. Evidence focusing specifically on nursing students in Saudi Arabia remains scarce, particularly in terms of how AI use interacts with study engagement and academic performance.

We identified important research gaps. To our knowledge, there is limited empirical work identifying distinct patterns of AI use among students using person-centered approaches such as latent class analysis. Only few studies integrate AI usage with core educational constructs such as study engagement and academic performance within a unified analytical framework. Evidence from Middle Eastern settings, particularly Saudi Arabia, also remains scarce despite rapid digital transformation and increasing adoption of AI technologies in higher education.

This study addresses these gaps by identifying latent profiles of AI use and study engagement among nursing students and examining their associations with academic performance. By applying a person-centered approach, integrating key educational constructs within a single framework, and generating evidence from the Saudi Arabian context, this study contributes new insights into how different patterns of AI-supported learning relate to educational outcomes. These findings may inform educators, curriculum developers, and policymakers seeking to promote effective and responsible use of AI in nursing education.

Therefore, we aim to identify latent profiles of AI use and study engagement among nursing students and to examine how these profiles are associated with academic performance within the Saudi Arabian context.

## 2 Methods

### 2.1 Study design and setting

We performed a cross-sectional survey design to examine patterns of AI use and learning engagement among undergraduate and postgraduate nursing students at King Saud University. The data were collected using google forms between February 11 and February 17, 2026. Recruitment was conducted through a convenience sampling approach. A QR code linking to the survey was displayed on official university announcement boards. Students voluntarily accessed the survey by scanning the QR code using their mobile devices. The first page of the questionnaire explains clearly what data will be collected, and students are asked specifically for their consent before proceeding.

### 2.2 Participants and eligibility

Participants eligible for inclusion were students undertaking training in nursing at the university during the course of the study. The participants included first-year preparatory students, undergraduate students in all academic levels, and graduate students at both Master's and PhD levels. Criteria of inclusion involved the participant being enrolled at the university and providing answers to questions concerning all variables under analysis by the latent class modeling. Any answers that had missing values on core behavior measures were excluded.

### 2.3 Measures

A formal a priori power analysis was not conducted because the study was exploratory in nature and aimed to identify latent profiles of AI use and study engagement. Sample adequacy was evaluated in relation to the complexity of the latent class model. The final sample comprised 254 participants and included a limited number of latent class indicators, which is generally considered adequate for estimating latent class models with a small number of classes and parameters.

AI use for learning purposes was assessed using four binary indicators representing common educational applications of generative AI tools. These included using AI for understanding concepts, summarizing content, drafting assignments, and language support. The indicators were selected to capture both content-related learning activities and task-oriented academic assistance that students commonly obtain from AI systems. Each item was coded as “Not selected” (0) or “Selected” (1). For latent class analysis, the variables were recoded to 1 and 2, with higher values indicating use of AI for the specified purpose. These indicators were treated as distinct behavioral measures of AI use rather than components of a single scale.

The learning efficiency was examined using five questions that addressed the extent to which participants felt they learned effectively. These included effective learning, understanding of subject matter, knowledge application, learning efficiency, and overall satisfaction. All questions were rated using a five-point Likert scale anchored between “not at all characteristic” and “entirely characteristic.” Learning scores were derived by taking the average of ratings obtained for each question. This variable was treated as continuous and used in regression and validation analyses but was not included in the latent class model.

Study engagement was assessed using selected items adapted from the Online Student Engagement Scale developed by Dixson (18), a widely used instrument with demonstrated content validity and good internal consistency in higher education settings. These items included behavioral engagement, including studying regularly, putting effort into coursework, keeping up with readings, and being organized. Responses were measured on a five-point Likert scale ranging from “Not at all characteristic” to “Entirely characteristic.” To improve model stability and reduce sparsity, we collapsed responses into three ordered categories: low (1, 2), moderate (3), and high (4, 5). These variables were included as indicators in the latent class analysis. Although categorization may result in some loss of information by reducing variability within response categories, the approach was considered appropriate given the sample size and the exploratory objective of identifying broader patterns of engagement behavior rather than fine-grained differences in response levels.

In this study, academic performance was evaluated using three self-reporting questions which were an index of performance during test-taking, academic grading, and academic course performance. These questions were rated on a five-point Likert scale, and their mean score was used to obtain a continuous variable. This continuous variable was used to validate differences between latent classes.

We also included several demographic variables, included age, gender, academic level, and grade point average (GPA). Age was categorized into five original groups and then collapsed into two categories to ensure sufficient sample size within each group (18–20 and 21+). Academic level was recoded into broader categories by combining preparatory and early undergraduate students into a “Junior undergraduate” group and grouping senior undergraduate and postgraduate students into an “Advanced (senior undergraduate/postgraduate)” category. GPA was categorized into ordered groups and further collapsed to reduce sparse categories < 3.0, 3.0–3.49, 3.5–3.99, 4.0–4.49, 4.5–5.0. Sex was treated as a binary variable (Male, Female).

### 2.4 Data preparation

As part of preparing the data for latent class analysis, variables were transformed into numerical forms from their labeled forms initially. Binary variables related to AI usage were transformed from a range of 0–1 to 1–2. Engagement level Likert scale variables were reduced to three categories. Participants with missing values on any of the latent class indicator variables were excluded using complete-case analysis. Of the 391 survey respondents, 137 (35.0%) were excluded because of missing information on one or more variables required for the latent class analysis, resulting in a final analytical sample of 254 participants.

Demographic variables were transformed into factors with a definite reference category.

### 2.5 Reliability and validity of study measures

Internal consistency of multi-item scales was assessed using Cronbach's alpha in the current sample. The Trust in AI scale demonstrated excellent reliability (Cronbach's α = 0.95), the Learning Effectiveness scale demonstrated excellent reliability (Cronbach's α = 0.98), and the Responsible Habit scale demonstrated acceptable reliability (Cronbach's α = 0.79). These values indicate satisfactory internal consistency of the study measures. Reliability was not assessed for the AI use indicators because they represent distinct learning behaviors rather than a single underlying construct.

### 2.6 Statistical analysis

#### 2.6.1 Latent class analysis

We employed a Latent Class Analysis (LCA) to extract unobserved clusters of students based on AI use and study involvement behaviors (19). In addition to binary indicator variables for AI use, an ordinal variable for study involvement was considered for the analysis. Models with up to five latent classes were fitted using Maximum Likelihood Estimation. In order to avoid convergence issues due to local maxima, each model was estimated using thirty starting values and a maximum iteration limit of 5,000. Model selection was done using various model comparison criteria, such as the Akaike Information Criterion (AIC), Bayesian Information Criterion (BIC), log-likelihood value, and Entropy.

Models with lower values for AIC and BIC criteria and higher values for entropy were deemed more favorable. However, in addition to these statistical factors, the interpretability of the resulting latent classes also played a significant role in the model selection process. Individuals were assigned to their respective latent classes using the posterior probability approach.

#### 2.6.2 Profile and validation analyses

We obtained class-level profiles through the conditional probabilities for use of AI variables and average values for engagement measures. The class-level profiles were then used to characterize each class. Class distributions were also computed as another means of describing the distribution of students within each class.

For external validation, differences in academic achievement between classes were determined using analysis of variance (ANOVA).

#### 2.6.3 Multinomial logistic regression

We performed a multinomial logistic regression to examine demographic predictors of latent class membership. The dependent variable was class membership. This was obtained based on posterior probability, where each participant was assigned to a single latent class with their highest posterior probability. The resulting categorical variable comprised three groups: “Strategic Engagers,” “Moderate Users,” and “Passive Users.” We used “Passive Users” group as the reference category, as it represented the lowest level of AI use and study engagement. Independent variables included age, gender, academic level, and GPA. All predictors were treated as categorical variables. Reference categories were selected based on theoretical relevance and distribution of the data. Results were reported as odds ratios with corresponding confidence intervals.

#### 2.6.4 Multiple linear regression

In order to determine whether latent class membership was related to effective learning, we performed a multivariable linear regression analysis using the learning score as the outcome variable. Latent class was used as the main predictor, with the “Passive Users” group set as the reference group. We have adjusted for several covariates including age group, gender, education level, and GPA, all being categorical variables. Regression coefficients together with their 95% confidence intervals were estimated.

Model assumptions were assessed. We assessed assumptions of linearity, normality of residuals, homoscedasticity, and multicollinearity were evaluated through residual plots, Q-Q plots, and variance inflation factors (VIFs).

All analyses were conducted using the R statistical environment (20). Key packages included poLCA for latent class analysis (21), nnet for multinomial regression (22), and ggplot2 for visualization (23).

## 3 Results

**Figure 1:**
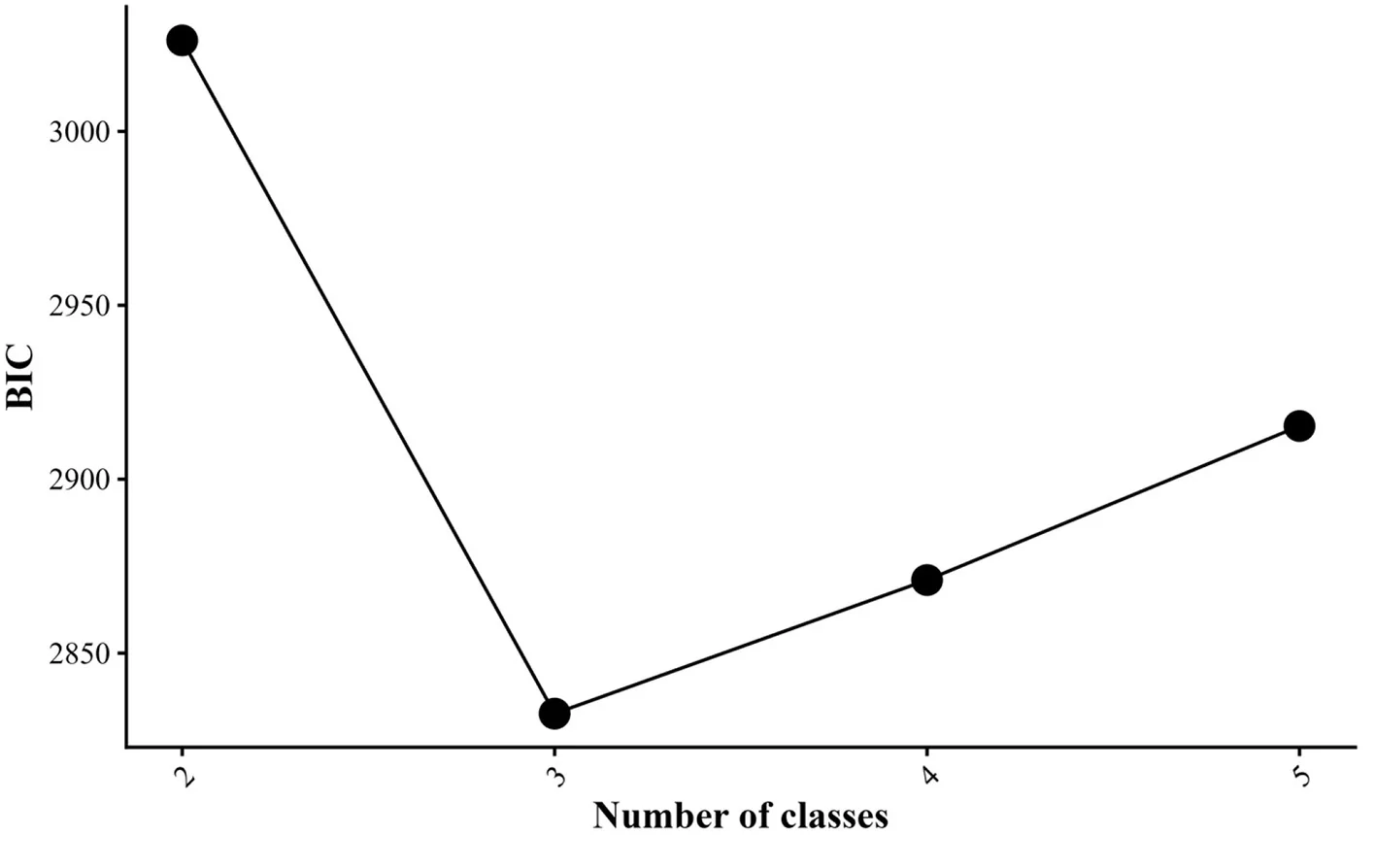
Bayesian Information Criterion (BIC) values for latent class models with two to five classes.

The total number of participants were 254. The latent class of variable distinguished between three subgroups based on students' AI usage and their engagement in studying: Strategic engagers (*N* = 116), moderate users (*N* = 63), and passive users (*N* = 75) (Table 1). The largest percentage of students in all three subgroups was aged between 21–23 and above, being 60% in both subgroups (strategic engagers and moderate users), whereas passive users made up 64%. In addition, the proportion of male respondents was very high in all subgroups. We also observed a consistent patter across academic level groups. Most participants were enrolled at advanced levels of study, including senior undergraduate or postgraduate programs, with proportions ranging from 83% among passive users to 89% among moderate users. However, there was high variance in the GPA between the three latent classes. The strategic engagers had a well-balanced GPA profile, which included greater representation in the top performance (4.5–5.0). Moderate and passive users had majority representation in the GPA (4.0–4.49). The Bayesian Information Criterion (BIC) reached its lowest value for the three-class solution, indicating that the three-class model provided the best balance between model fit and parsimony (Figure 1)

**Table 1:** Distribution of sociodemographic and academic characteristics across latent classes of AI use and study engagement (Strategic Engagers, Moderate Users, and Passive Users).

Characteristic	Strategic engagers *N* = 116	Moderate users *N* = 63	Passive users *N* = 75
Age			
18–20	46 (40%)	25 (40%)	27 (36%)
21–23+	70 (60%)	38 (60%)	48 (64%)
Sex			
Female	40 (34%)	22 (35%)	25 (33%)
Male	76 (66%)	41 (65%)	50 (67%)
Academic_level			
Junior UG	14 (12%)	7 (11%)	13 (17%)
Advanced (Sr UG/PG)	102 (88%)	56 (89%)	62 (83%)
GPA			
< 3.0	15 (14%)	5 (8.9%)	6 (8.7%)
3.0–3.49	20 (19%)	8 (14%)	17 (25%)
3.5–3.99	24 (23%)	12 (21%)	11 (16%)
4.0–4.49	24 (23%)	26 (46%)	31 (45%)
4.5–5.0	22 (21%)	5 (8.9%)	4 (5.8%)
Unknown	11	7	6

In the latent class analysis, we identified three distinct behavioral profiles based on patterns of AI use and study engagement (Table 2). These classes showed that the difference was more strongly in engagement behaviors than in AI use. This means the difference was rather on how students study, rather than whether they use AI. The largest group, labeled strategic engagers (45.7%), demonstrated consistently high levels of study engagement across all indicators. Mean scores approached the upper range for study regularity (2.80), effort (2.91), engagement with required readings (2.72), and organization (2.82). In contrast, their use of AI tools was selective and purpose driven. They showed relatively high probabilities of using AI for concept understanding (0.76) and summarization (0.65), but lower reliance on drafting (0.22) and use for language support (0.42). Our finding suggests that these students might rather integrate AI as a cognitive support tool than a substitute for active learning.

**Table 2:** Latent class profiles of AI use and study engagement among nursing students, with conditional probabilities of AI learning purposes and mean scores of study engagement behaviors across identified classes.

AI purposes (probabilities)					Study engagement (mean scores)				
Latent class persona	Concept understanding	Content summarization	Draft generation	Language support	Study regularity	Study effort	Required readings	Study organization	*n* (%)
Strategic engagers	0.76	0.65	0.22	0.42	2.80	2.91	2.72	2.82	116 (45.7)
Moderate/utility users	0.79	0.68	0.30	0.44	2.02	1.98	1.75	2.00	63 (24.8)
Passive/low engagers	0.79	0.67	0.28	0.55	1.04	1.04	1.13	1.13	75 (29.5)

The moderately engaged group (24.8%) showed moderate engagement with regard to all the aspects associated with their learning habits, having average scores of 2.0 for all three dimensions of engagement (regularity, effort, and organization), and 1.75 for reading assignments. In their use of artificial intelligence, the moderately engaged students showed similarities with strategic engagers when it comes to their understanding (0.79) and summarization (0.68), though they made slightly greater use of drafting (0.30).

**Figure 2:**
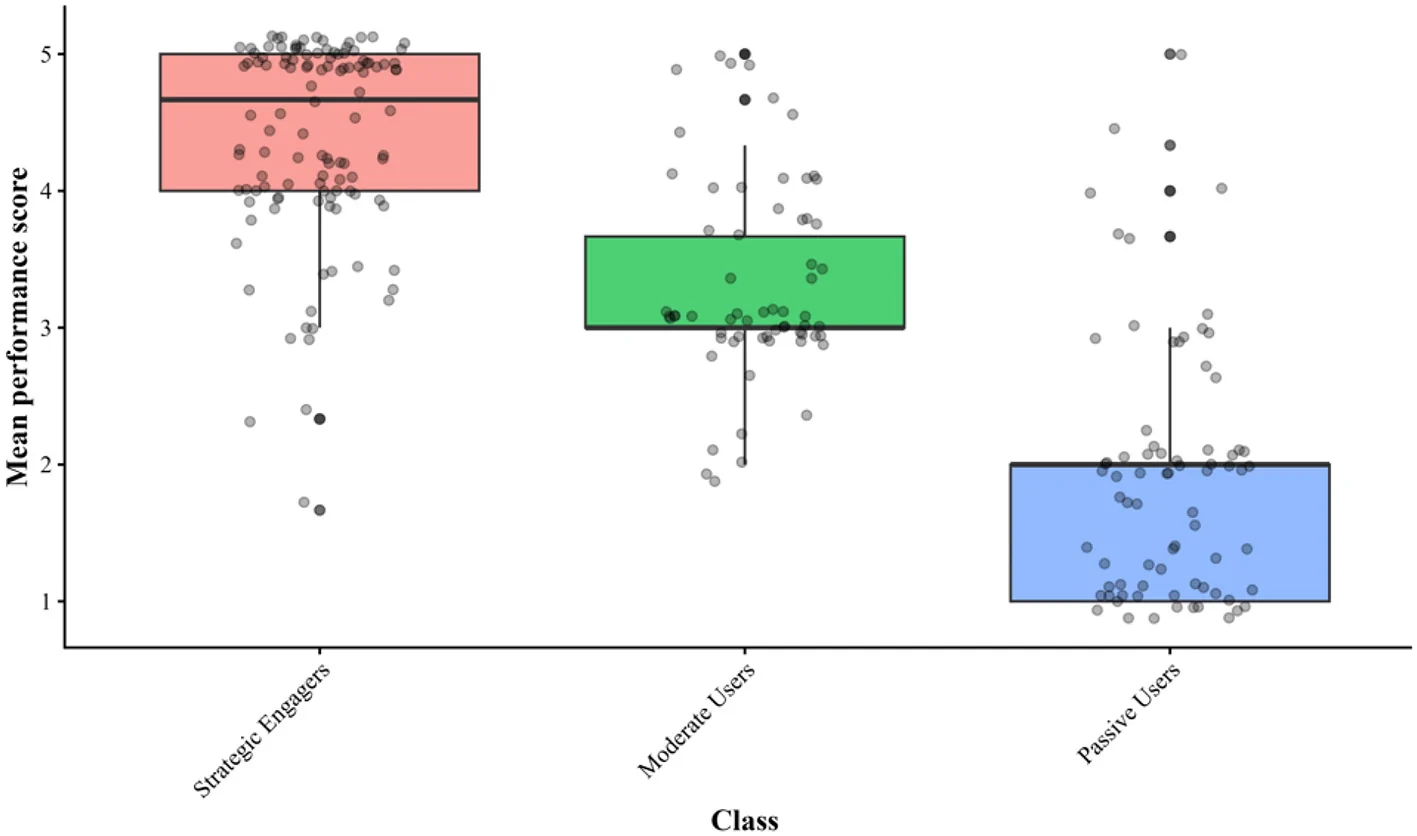
The distribution of AI use and study engagement using mean performance score.

**Figure 3:**
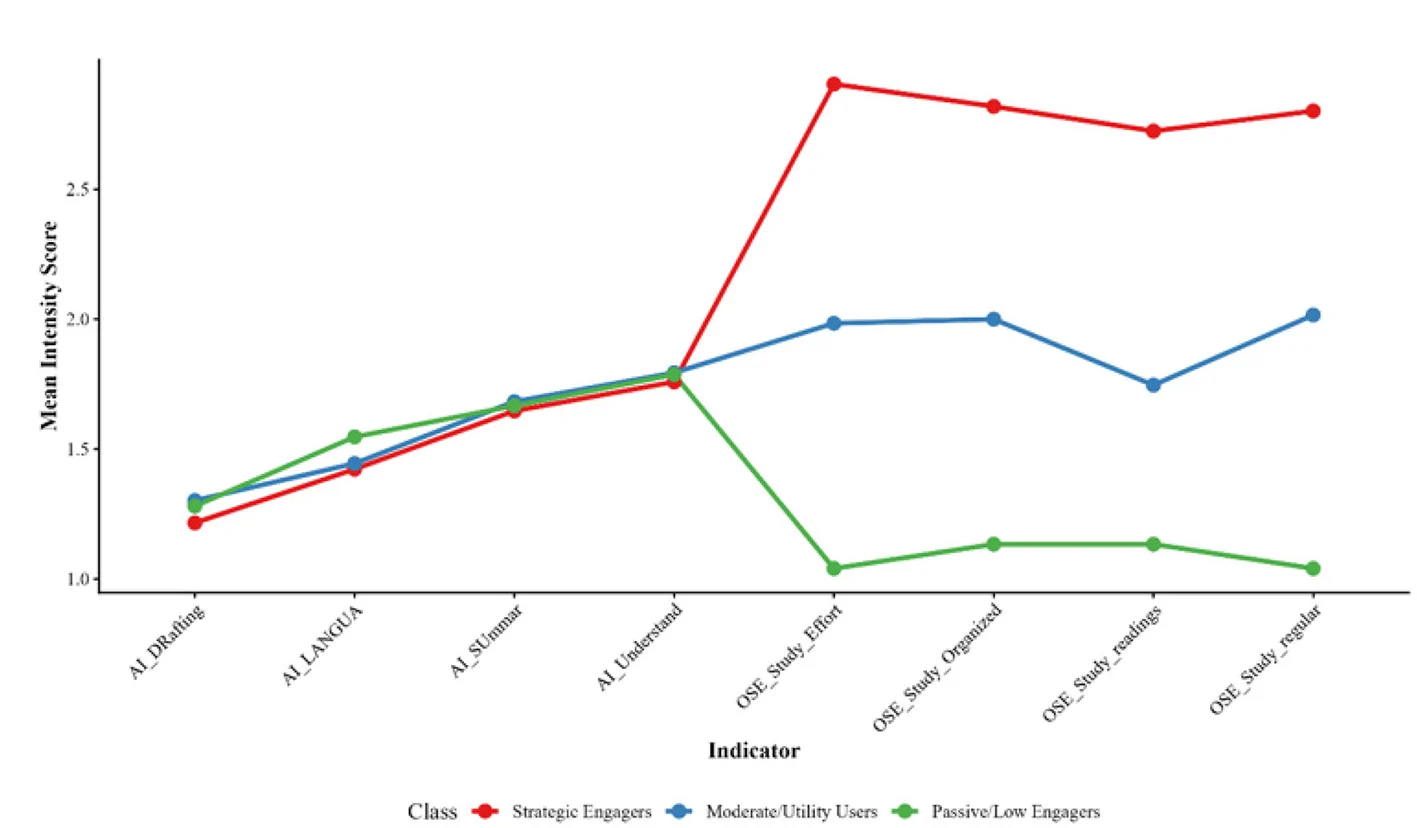
Mean intensity score across indicators.

Passive engagers (29.5%) had consistently low engagement in all aspects of study behavior, such that their mean scores were at the bottom level in regard to regularity (1.04), effort (1.04), readings (1.13), and organization (1.13). However, this did not affect their utilization of AI tools since it was relatively high in comparison to other segments. In particular, this group had the highest probability of employing AI tools for language (0.55), understanding (0.79), and summarization (0.67). The mean performance score was also varies across the identified latent classes (Figure 2). The identified latent classes showed similar patterns of AI use but were clearly differentiated by study engagement, with Strategic Engagers consistently exhibiting the highest engagement levels, Moderate/Utility Users showing intermediate levels, and Passive/Low Engagers demonstrating the lowest engagement (Figure 3).

The results from multinomial logistic regression examining the association between demographic and academic factors and latent class membership was summarized in Table 3.

**Table 3:** Associations of latent class membership and demographic characteristics with learning effectiveness and class membership among nursing students.

Predictor	Multiple linear regression	Multinomial logistic regression	
	β (95% CI)	Strategic engagers OR (95% CI)	Moderate users OR (95% CI)
User class (Ref: passive users)			
Strategic engagers	2.39 (2.08, 2.69)		—
Moderate users	1.22 (0.87, 1.56)	—	—
Age (Ref: 18–20 years)			
21–23+ years	−0.02 (−0.28, 0.24)	0.75 (0.39, 1.47)	0.77 (0.36, 1.64)
Sex (Ref: female)			
Male	0.01 (−0.28, 0.31)	0.80 (0.38, 1.71)	1.16 (0.50, 2.67)
Academic level (Ref: Junior UG)			
Advanced (Sr UG/PG)	−0.02 (−0.40, 0.36)	1.49 (0.59, 3.78)	1.51 (0.51, 4.51)
Academic GPA (Ref: < 3.0)			
3.0–3.49	−0.20 (−0.67, 0.27)	0.45 (0.14, 1.45)	0.58 (0.13, 2.51)
3.5–3.99	−0.05 (−0.52, 0.42)	0.80 (0.24, 2.70)	1.33 (0.31, 5.73)
4.0–4.49	0.29 (−0.17, 0.75)	0.26 (0.08, 0.83)^*^	1.01 (0.26, 3.96)
4.5–5.0	0.05 (−0.47, 0.58)	1.82 (0.41, 8.02)	1.50 (0.24, 9.29)

Results are presented as regression coefficients (β) with 95% confidence intervals from multivariable linear regression, and odds ratios (OR) with 95% confidence intervals from multinomial logistic regression. In the multinomial model, the reference category was “Passive Users.” In the linear regression model, learning effectiveness was treated as a continuous outcome.

There was no significant association between age group and class membership. Students aged 21 years and older did not have significantly different odds of being classified as strategic engagers (OR = 0.75, 95% CI: 0.39–1.47) or moderate users (OR = 0.77, 95% CI: 0.36–1.64) compared with those aged 18–20 years. Similarly, no significant association was observed for sex. Male students had comparable odds of being strategic engagers (OR = 0.80, 95% CI: 0.38–1.71) and moderate users (OR = 1.16, 95% CI: 0.50–2.67) relative to female students. The wide confidence intervals indicate substantial uncertainty around these estimates. We also found no significant association between academic level and class membership. Students in advanced academic levels had similar odds of belonging to the strategic engager (OR = 1.49, 95% CI: 0.59–3.78) and moderate user classes (OR = 1.51, 95% CI: 0.51–4.51) compared with junior undergraduate students. Compared with students with a GPA below 3.0, those with a GPA of 4.0–4.49 had significantly lower odds of being classified as strategic engagers rather than passive users (OR = 0.26, 95% CI: 0.08–0.83). No significant associations were observed for the remaining GPA categories or for membership in the moderate user class.

There was significant association between latent class membership and learning effectiveness. We found that, compared to passive users, students classified as strategic engagers demonstrated substantially higher learning scores (β = 2.39, 95% CI: 2.08–2.69. Similarly, those categorized as moderate users also showed higher learning scores compared with passive users (β = 1.22, 95% CI: 0.87 to 1.56). No significant association was found for demographic characteristics.

Diagnostic assessment indicated no major violations of the regression assumptions (Supplementary Figure S1). Residuals were approximately normally distributed, variance was reasonably constant across fitted values, and no highly influential observations were detected.

## 4 Discussion

In this study, three different student profiles were detected according to their behavioral patterns in using AI and engaging in studies, and it was found that the identified profiles were highly related to learning effectiveness. Although the application of AI in studying was common and almost identical among all profiles, there were some significant differences regarding study engagement behavior. Almost one-half of the respondents were regarded as strategic engagers due to their consistent efforts, regular study routine, and effective organization skills, while the other two groups were moderate and low engagement levels, respectively. These behavioral differences were associated with a clear gradient in learning effectiveness, with strategic engagers achieving substantially higher learning outcomes than both moderate and passive groups, and moderate users also outperforming passive users.

Our findings are consistent with several previous studies that focused on central role of student engagement in academic success. A meta-analysis demonstrated that behavioral, cognitive, and emotional engagement are consistently associated with improved academic performance across educational settings (24). Our findings extend this literature to AI-supported learning environments by suggesting that engagement remains a key differentiating factor even when AI use is widespread among students. This observation is particularly important because it challenges the assumption that access to advanced learning technologies alone is sufficient to improve educational outcomes. Instead, AI appears to be most beneficial when embedded within active and organized learning behaviors (24). Studies also showed that online and blended learning environments report similar findings, where engagement indicators outperform technology (25, 26). Our findings also clearly show that engagement differentiates high performing students that AI use

In our findings, the limited variation in the use of AI across latent class is important. All groups reported relatively high probabilities of using AI for understanding concepts and summarizing content, suggesting that AI tools are widely adopted among nursing students. This finding was consistent with several cross-sectional studies in higher education (27, 28). However, these studies highlighted that AI use does not necessarily lead to improved performance, which can be due to accuracy, privacy and bias. Our findings suggest that students with similar patterns of AI use may differ in learning effectiveness according to their levels of study engagement. The findings of this study are consistent with an experimental study that found removing AI use from students decreases their performance more than those who never used AI (29). In our study, we did not find significant association between demographic characteristics and class membership. Recent evidence suggests that nursing students generally demonstrate favorable attitudes toward AI and increasing readiness to incorporate AI tools into their educational experiences (30). Within this context, variation in AI adoption may become less important than variation in how students engage with learning while using these technologies. This observation has important implications for the conceptual framework of the study. We initially anticipated that distinct patterns of AI use might differentiate student profiles and contribute to differences in learning effectiveness. However, the results suggest that AI adoption was widespread across all groups, whereas engagement behaviors were the primary factors distinguishing the profiles. This finding indicates that access to or use of AI tools alone may not be sufficient to explain differences in learning outcomes. Instead, the educational value of AI may depend on how students incorporate these tools within broader learning strategies and study behaviors

We found that students with GPA 4.0–4.49 were less likely to belong to the strategic engagers, which might be counterintuitive. In contrast, previous studies typically report positive associations between academic achievement and effective learning behaviors (31–33). There are several justification for our findings. It is possible that higher-achieving students adopt more efficient, outcome-oriented learning strategies rather than high-intensity engagement behaviors. One possible explanation is that some higher-achieving students rely more heavily on prior knowledge or employ more efficient learning strategies that were not captured by the engagement measures used in this study

### 4.1 Implications for nursing education, policy and future research

The findings show that learning outcomes depend on study engagement rather than AI use alone. It is necessary to incorporate AI into the existing system of teaching techniques. Learning should focus on clinical reasoning, AI critical analysis, and application of what one learns through practical approaches. Using AI in activities such as problem-based learning, simulation, and reflection may ensure its incorporation to aid professional reasoning, and not substitute it.

Our findings suggest that educational policies may benefit from focusing not only on regulating AI use but also on promoting effective and ethical integration of AI into learning activities. There is a need for clear policies that define what is considered acceptable in terms of usage and academic honesty.

Future research should adopt a longitudinal design to assess changes in the use and engagement with AI tools and their impact on learning outcomes. It would be necessary to incorporate measures of academic performance and additional variables pertaining to the use of AI tools, including the frequency and intensity of usage. Intervention studies can also be conducted to assess engagement techniques.

### 4.2 Strength and limitations

This study has several important strengths. We employs a person-oriented analysis strategy with latent class analysis, thus allowing the identification of specific behavior patterns instead of depending on mean effects. This gives a more detailed look at how learners integrate their usage of AI technology with engagement. The combination of latent class analysis with regression models increases the effectiveness of analysis through the connection between behavior types and learning outcomes and demographic variables. Another strength of this study is that it involves several domains of measurement, such as AI usage, engagement, and performance. It avoids focusing on just one aspect of learning. Content validity is ensured due to the employment of existing measures of engagement and standardized assessment of usage frequency. The contribution of this work lies in its novelty since there is very little evidence on AI usage in education in the Middle Eastern region.

Several limitations should be considered when interpreting the findings. Cross-sectional designs preclude the ability to make causal claims, while the correlations obtained cannot establish any temporal relationship among the use of AI tools, engagement, and academic performance. Moreover, since all measures used were self-reported, there might be a chance that participants were subject to recall bias or social desirability bias when answering questions about their academic performance or engagement. The measurement of academic performance relied on perception-based scores rather than on actual grades, which could have impacted the validity of the measure. Measures of the use of AI were only able to establish whether participants picked a certain purpose, without accounting for frequency, intensity, or quality of use, which could have limited their discriminative power and the ability to distinguish between the classes. There were several indicators whose dispersion was low, implying the likelihood of ceiling effects in the adopted AI use levels. However, collapsing Likert-scale engagement measures into three levels enhanced model stability. The data set comprised observations from only one institution obtained by way of convenience sampling using a QR-code invitation. Furthermore, the study was conducted at a single university in Saudi Arabia, which may limit the generalizability of the findings to nursing students in other institutions, educational systems, or cultural contexts. Therefore, the identified profiles and their associations with learning effectiveness should be interpreted cautiously. Lastly, while LCA is a highly informative technique, class membership probabilities could pose challenges to the results of subsequent regression analyses.

## Data Availability

The raw data supporting the conclusions of this article will be made available by the authors, without undue reservation.
