# Supplementary material for: Digital personas of AI use in nursing education: a latent class analysis of learning behaviors and academic engagement
Source: Front Med (Lausanne). 2026 Aug 14;13:1859093. doi: 10.3389/fmed.2026.1859093 (PMC13521864; doi:10.3389/fmed.2026.1859093)
Supplement: Supplementary file 1 [file Table_1.docx]

**Supplementary Materials**

**Digital Personas of AI Use in Nursing Education: A Latent Class Analysis of Learning Behaviors and Academic Engagement**

Majed M. Aljabri^1*^, Bandar S. Alharbi^1^, Endale Alemayehu Ali^2^*

^1^Community and Psychiatric Mental Health Department, College of Nursing, King Saud University, Riyadh 12375, Saudi Arabia

^2^Department of Public Health and Primary Care, KU Leuven, Kapucijnenvoer 33, 3000 Leuven, Belgium

^*^ Shared corresponding author (Email: [amajad@ksu.edu.sa](mailto:amajad@ksu.edu.sa); [endalestat@gmail.com](mailto:endalestat@gmail.com))

Table S1: Model Fit Indices for Latent Class Solutions

| Classes | AIC | BIC | LogLik | Entropy |
| --- | --- | --- | --- | --- |
| 2 | 2,937.73 | 3,026.16 | -1,443.86 | 0.96 |
| 3 | 2,698.20 | 2,832.62 | -1,311.10 | 0.94 |
| 4 | 2,690.62 | 2,871.02 | -1,294.31 | 0.89 |
| 5 | 2,688.91 | 2,915.30 | -1,280.46 | 0.92 |

Figure S1. Diagnostic plots for assessment of multiple linear regression assumptions
